# Pain Management in Laparoscopic Donor Nephrectomy: A Review

**DOI:** 10.1155/2012/201852

**Published:** 2012-10-23

**Authors:** U. Mathuram Thiyagarajan, A. Bagul, M. L. Nicholson

**Affiliations:** Transplant Surgery Group, Department of Infection, Immunity and Inflammation, Leicester General Hospital, University of Leicester, Gwendolen Road, Leicester LE5 4PW, UK

## Abstract

The management of postoperative pain is a key to patient early recovery, in particular, where the surgery was performed to benefit another human being. In recent years it has been recognized that multimodal analgesic methods are superior for postoperative pain relief. It is also imperative to remember that inadequately managed acute postoperative pain opens the doorway to possible suffering from chronic postoperative pain later. Although the laparoscopic donor nephrectomy has reduced the disincentives associated with open surgery, still significant percentage of donors suffers from postoperative pain. In the UK, patient-controlled analgesic system (PCAS) using morphine for postoperative pain relief is being used in majority of the transplant centres. Though opioids provide good analgesia, they are far from being an ideal analgesic due to their adverse effects. This paper pragmatically looks in depth on different modalities of pain management in patients undergoing laparoscopic live donor nephrectomy.

## 1. Introduction

The donor nephrectomy is a procedure carried out to benefit another individual and in addition it can add a lot of disincentives to the donor. Subjecting a patient to an open operation leads to increased hospital inpatient stay and a much more painful large scar, thus not only discouraged the potential donors, but also it leads to increased morbidity for long time. This has stimulated the surgeons to come up with an alternative, the laparoscopic donor nephrectomies.

The first laparoscopic live donor nephrectomy (LLDN) was performed by Ratner et al. [1] at the Johns Hopkins Bay view Medical Center, Baltimore, USA in February 1995. The donor was discharged on first postoperative day and returned to work 2 weeks later. This technique thus revolutionized the donor nephrectomy and also removed the added disincentives of open operation.

LLDN is now the preferred method and gold standard operation for kidney donation. Although the LLDN is associated with the longer operation time, it has reduced morphine requirement, hospital stay, and postoperative complications with an early return to work [2]. Randomized controlled trials and systematic reviews confirmed that LLDN is safe and reduce the morbidity following the operation [2–4]. 

## 2. Pain after LLDN

Pain following the LLDN is multifactorial. Port pain, low abdominal incisions (to retrieve the kidney), pelvic organ nociception, diaphragmatic irritation (shoulder tip discomfort from residual pneumoperitoneum), urinary catheter discomfort add-up and contribute to the total pain experience.

Several studies confirmed that laparoscopic and hand-assisted nephrectomies produce less pain compared with an open operation [5–9]. Nonetheless, some patients undergoing laparoscopic live donor nephrectomy still suffer significant postoperative pain, to the point where they require parenteral opioids. Based on the assumption that minimally invasive approaches are less traumatic, some units avoid opioids and neuraxial techniques [10].

Nevertheless, LLDN can cause severe neuropathic pain possibly by nerve lesions caused by trocars [11]. The aim of this paper is to create an evidence-based document reviewing the current literature with a view to address the best, possible pain relief methods in laparoscopic donor nephrectomy patients.

## 3. Postoperative Pain and Its Implications

Pain has a wide spectrum of effects on the body. Inadequately controlled postoperative pain may have harmful physiologic, psychological consequences which potentially increases the morbidity and mortality [12, 13].

 It has been recognized that inadequately treated postoperative pain may lead to chronic pain which is often misdiagnosed and neglected [10, 14]. The significance of this association has been confirmed in other studies on a healthy patients undergoing caesarian section [14] and in patients after inguinal hernia repair [15]. International Association for Study of Pain (IASP) defines the chronic postsurgical pain (CPSP) as pain lasting more than 6 months for nontumour cause and more 3 months in malignancy [16]. Dillenburg et al. [5] found that 20% patients reported CPSP 6 months after nephrectomy. Similar high incidence of CPSP had been shown after open donor nephrectomy in other studies as well [17, 18]. In our centre, we have reported 5% chronic pain in patients undergoing LLDN [19]. 

The chronic persistent pain after surgery can be caused by many factors but most notably the severity of postoperative pain and psychologic vulnerability. Patients with higher severity of postoperative pain (particularly movement evoked pain-dynamic pain) are more likely to have chronic pain [20–26]. Hence an adequate dynamic pain relief protocol may reduce the development of chronic pain after surgery.

The multimodal analgesic methods have been shown to control this dynamic pain. Opioids are potent analgesics but are mostly inadequate to treat such dynamic pain [27–29], while local anesthetics methods, NSAIDS, *α*_2_ agonists, and NMDA receptor antagonists may be important for controlling dynamic type of pain and also preventing central sensitization [23–26].

## 4. Multimodal Approach

### 4.1. NSAIDS

Nonsteroidal anti-inflammatory drugs (NSAIDS) are generally avoided because of their potential nephrotoxicity and other adverse effects. NSAIDS are found to have little effect on surgical stress response and organ dysfunction [30, 31]. But on the other hand, it has been shown that NSAIDS provide moderate postoperative analgesia and thereby an opioid sparing effect in about 20–30% [18]. Hence they can reduce the incidence of opioids-related adverse effect like nausea, vomiting, respiratory depression, ileus, and bladder disturbances. If NSAIDS are used for less than 5 days with adequate hydration, they can make a potential alternate to opioids.

Freedland et al. [32] used Ketorolac in LLDN patients and noted no differences in renal function. Patients who underwent surgery after introduction of Ketorolac-based analgesia had a significantly shorter postoperative stay. But Ketorolac has been associated with serious side effects including gastrointestinal bleeding, postsurgical bleeding, and impairment of renal function, particularly when used for more than 5 days [33–35]. This hesitates transplant units to widely use the NSAIDS in donor nephrectomy patients.

### 4.2. Opioids Analgesia

The use of morphine in the postoperative period is a standard practice. Morphine can be given either as an intramuscular/intravenous bolus or through a patient-controlled analgesia system (PCAS). Although PCAS system is widely used, we do not know the best way of morphine administration. Some studies found PCAS as preferred [36, 37], but others could not replicate similar results [38, 39]. The patient satisfaction is found to be more with the use of PCAS [40] with less nursing time [41]. Recent literature review found that patients getting intramuscular morphine were associated with the higher rate of inadequate analgesia exposure/experience [42].

PCAS does not appear to provide optimal dynamic pain relief after a major surgery [42] Meta-analysis [40] and randomised control trials [41, 43–46] have also shown that postoperative morbidity is not reduced by PCAS compared to intermittent morphine opioids. These findings are consistent with the lack of effect on PCAS on surgical stress response and organ dysfunction [30, 31]. In addition, high incidences of postoperative nausea/vomiting (PONV), respiratory depression and sedation are noted in morphine use when compared to epidural analgesia [42].

Hence making the use of opioids far from being the ideal postoperative analgesics of choice following a major surgery like LLDN.

### 4.3. Epidural Analgesia

Usually this technique is used as a substitute for PCAS in LLDN patients. Epidural opioids and local anaesthetics infiltrations are known to provide more effective dynamic pain relief [47]. But it is worth to note that epidural opioids are less effective on stress response [47]. Continuous epidural administration of local anaesthetic (LA) agents or LA plus opioids has been shown to reduce the postoperative pulmonary morbidity after major abdominal surgery [48]. They can also block the sympathetic responses and may reduce the cardiac morbidity [13]. Epidural analgesia is found to be associated with a lower incidence of PONV, sedation, and postoperative bowel dysfunction when compared with opioids [42].

But epidural analgesia has its own problems like urinary retention and risk of infection at the catheter site. Urinary retention is a common problem, and 23% of patients undergoing an epidural needed a urinary catheterization. Although it is one of the best modality of analgesia, its efficacy in major abdominal procedures is somewhat small because of the insufficient afferent neural blockade [47].

A retrospective series on a cohort of open donor nephrectomy has showed that thoracic epidural analgesia is better than a lumbar [49]. Though epidural analgesia provides good pain relief, it is associated with a lot of complication including nausea (47%), vomiting (22%), hypotension (11%), lower extremity motor blockade (8%), pruritus (5.5%), and somnolence (5%) [49]. Although epidural analgesia provides good pain relief, a thorough literature review cannot confirm a single study in LLDN patients.

### 4.4. Neuraxial Techniques

Blockade of afferent neural stimulus by local anaesthetic agents is very effective in reducing the classical catabolic responses to the surgery [42, 50, 51]. Thus the unusual increase of cortisol, catecholamines, and glucose concentration can be prevented, insulin resistance reduced, glucose and nitrogen economy improved [42, 50, 51]. The unfavorable changes in the coagulatory-fibrinolytic systems are also modified in favour of less thrombosis formation, while most changes in the immune function and markers of inflammation remain unaltered by a neural block and concomitant hormonal inhibition [42, 50, 51]. It is also worth to note that pain relief by other techniques such as epidural analgesia, systemic opioids, NSAIDS is less effective than a normal block with local anaesthetic [50, 51]. Opioids are effective at the transmission stage, whereas pre-emptive local anesthetic and peripheral nerve blockade takes effect by preventing conduction of the nociceptive stimulus as well as preventing central sensitization [20, 52].

The subfascial administration of local anesthetic has been shown to be much more effective than subcutaneous injection in reducing the pain [52]. Subfascial administration of bupivacaine (0.5%) at the trocar and incision sites not only reduced the pain, but also they shortened the hospital stay in patients undergoing a laparoscopic nephrectomy [50].

### 4.5. Transversus Abdominis Plane (TAP) Block

Since the arrival of a TAP block technique in 2001 [53], it has been widely used for postoperative pain relief. Previous randomised controlled trials have shown that TAP block can reduce postoperative pain and morphine requirement after abdominal surgery for large bowel resection [54] and caesarean section [55]. The later study is particularly relevant as caesarean section is performed through a suprapubic Pfannenstiel incision, which is similar to the approach used for retrieving kidneys that have been dissected laparoscopically. 

The preemptive TAP block can potentially reduce the metabolic responses during surgery and also avoid the central sensitization. The principle behind preemptive TAP blocking is that local anaesthetic is injected into the neurofascial plane where it may act on the afferent sensory nerves of the lower 6 thoracic and upper lumbar nerves as they course through the plane before they pierce the musculature to innervate the abdominal wall. This plane is poorly vascularized, and it has been suggested that the prolonged analgesic effect can be observed in TAP blocking, due to slow drug clearance [54, 55].

#### 4.5.1. TAP Block Technique

With the patient in the supine position, a 22 gauge 50 mm blunted regional anesthesia needle was introduced laterally and posterior to the midaxillary line between the iliac crest and the inferior extent of the rib cage. The ultrasound probe is placed in transverse to the long axis of the abdomen and the needle is introduced perpendicular to the linear array beam of ultrasound. 

The presence of fascial extensions of the abdominal wall muscles was also used to correctly place the needle tip using the loss of resistance technique. The needle was held perpendicular to the coronal plane and advanced until resistance was encountered and a first “pop” sensation was felt. This indicated that the needle entered the plane between the external and internal oblique layers. The needle was then further advanced until a second “pop” sensation was encountered, indicating that the needle tip had traversed the fascial extensions of the internal oblique and was thus within the transversus abdominis plane.

To date this technique has not been incorporated in to laparoscopic donor nephrectomy patients; thus warrants and justifies a randomised control study to assess its efficacy. 

## 5. Continuous Infusion on Local Anesthetic Agents

As we know that local anesthetic agents can provide a lot of benefits without any systemic adverse effects. Recently Biglarnia et al. [56] showed the benefit of continuous infusion of Ropivacaine (0.5%) as a tool for postoperative pain relief. But here the donor nephrectomy was done through a hand-assisted retroperitoneoscopic technique (HARS). Two catheters were placed, one in the retroperitoneal space and another in the rectus sheath followed by continuous infusion of ropivacaine into both these spaces. This technique has dramatically reduced pain scoring and cumulative consumption of morphine equivalent.

Panaro et al. [57] have shown that continuous infusion of ropivacaine is a good pain relief technique for laparoscopic donor nephrectomy patients. In this study, retroperitoneal approach was used during the donor nephrectomy. Two catheters were used; one in between parietal peritoneum and muscle layers; second catheter was placed on subcutaneous tissue. This study also showed reduction in the pain score, morphine consumption, and hospital stay compared to the counterpart controls.

Unfortunately similar technique has not been used in transperitoneal LLDN patients. The use of similar technique in transperitoneal approach would be difficult; leaving the catheter for long in peritoneal cavity which also carries the risk of introducing infection and migration.

## 6. Long-Acting Local Anesthetics Agents

The long-acting local anesthetic agents do play an important role in postoperative pain relief. The prospect of long-acting local anesthetic agents that last for long is ideal and attractive but still under investigation in clinical trials. But preemptive portsite infiltration can reduce the central sensitization, facilitates recovery by enabling earlier ambulation [58–60], and may reduce the postoperative analgesics requirement. However, it is most effective for superficial procedures and analgesia which lasts for only 6–8 h.

Simple administration of local anesthetic after the cholecystectomy also reduces the right upper quadrant and shoulder pain [61, 62]. Although preincisional infiltration reduces the postoperative pain after cholecystectomy, [63–65] other investigators reported showed better pain relief when local anesthetic was infiltrated at the end of surgery [66]. Hence the local anesthetic infiltration at trocar site is still controversial [67].

The pain from suprapubic Pfannenstiel incision during the LLDN could be relieved by local anesthetic wound infiltration. Recent studi showed that local anesthetic infiltration before and/or after abdominal hysterectomy does not reduce the intensity of postoperative pain and analgesic requirements [68, 69]. But another randomized study proves benefit preemptive administration lidocaine efficient mode to reduce pain in the first eight hours after hysterectomy [70]. To date no study has been done in LLDN setting to assess the effect of long-acting local anesthetic agents for postoperative pain relief.

## 7. Other Methods

The acetazolamide can decrease the formation of H^+^ ions thereby retard peritoneal acidification which is probably responsible for visceral and referred pain [71]. Harvey et al. [72] have shown that the intravenous use of acetazolamide reduces referred pain following laparoscopic cholecystectomy.

Singh et al. [73] reported the beneficial use of acetazolamide as a part of multimodal analgesic approach in laparoscopic live donor nephrectomy. In this randomised double-blinded control trial, nasogastric administration of acetazolamide that has been used in combination of bupivacaine (0.5%) installation into renal fossa with (0.25%) infiltration at ports and retrieval wound was shown to be effective. The patients who received this multimodal therapy experienced less shoulder tip pain, pain scores at 12 hrs, total analgesic requirement with less nausea compared to controls. This study though was not powered to detect the drug-related side effects, it is the first documented study to test acetazolamide role in multimodal analgesia. The role of acetazolamide as a part of multimodal analgesia needs to be researched further.

## 8. Conclusion

The journey of finding the ideal method for pain relief in LLDN patients is yet far from being over. But the arrival of new techniques and multimodal approaches appears to be safe and effective on providing postoperative analgesia. The TAP block technique has not been tested in LLDN patients thus warrants a randomised control trial. 
